# *In vivo* molecular imaging of the neuroinflammatory response to peripheral acute bacterial infection in older patients with cognitive dysfunction: A cross-sectional controlled study

**DOI:** 10.3389/fnagi.2022.984178

**Published:** 2022-09-07

**Authors:** Ana Rita Silva, Patricia Regueira, André Peres, Ana Luísa Cardoso, Inês Baldeiras, Isabel Santana, Joaquim Cerejeira

**Affiliations:** ^1^Faculty of Psychology, Center for Research in Neuropsychology and Cognitive and Behavioral Intervention, University of Coimbra, Coimbra, Portugal; ^2^Center for Innovative Biomedicine and Biotechnology, University of Coimbra, Coimbra, Portugal; ^3^Department of Psychiatry, Centro Hospitalar e Universitário de Coimbra, Coimbra, Portugal; ^4^Proaction Lab, Faculty of Psychology and Educational Sciences, University of Coimbra, Coimbra, Portugal; ^5^Faculty of Medicine, University of Coimbra, Coimbra, Portugal; ^6^Department of Neurology, Centro Hospitalar e Universitário de Coimbra, Coimbra, Portugal

**Keywords:** neuroinflammation, acute systemic infection, delirium, dementia, [^11^C]-PK11195 PET

## Abstract

**Introduction:**

Chronic neuroinflammatory events have been implicated in the pathophysiology of neurodegenerative conditions but no studies have directly examined the neuroinflammatory response to acute systemic infection in older people with dementia. The objective of this study was to determine the magnitude of the neuroinflammatory response triggered by acute systemic infection in older subjects with dementia and/or delirium compared to cognitively healthy controls.

**Methods:**

We recruited 19 participants (4 with delirium, 4 with dementia, 4 with delirium superimposed on dementia, 7 cognitively healthy) hospitalized with acute systemic bacterial infection not involving the Central Nervous System. Participants underwent [^11^C]-PK11195 PET and a neuropsychological assessment during hospital stay. The distribution volume ratio was estimated in the regions-of-interest using the Hammers’ brain atlas.

**Results:**

In the subcortical analysis, we found that the cognitively healthy group presented regions with significantly higher DVR intensity than the other groups in the choroid plexus. Mean choroid plexus DVR positively correlated with MoCA (*r* = 0.66, *p* = 0.036).

**Conclusion:**

This study suggests that dementia and/or delirium is associated with a reduced neuroinflammatory response to acute systemic bacterial infection which can be the result of an immunosuppressive brain environment.

## Introduction

Dementia, including Alzheimer’s Disease (AD), is a known risk factor for acute systemic infections not involving directly the brain (e.g., pneumonia or urinary tract infection) which are also a common precipitant factor of delirium in this population (van der Steen et al., 2007). Chronic neuroinflammatory events are known to play an important role in dementia, particularly in AD. In fact, one of the hallmarks of AD is presence of activated microglia and reactive astrocytes in Aβ plaques and neurofibrillary tangles together with a broad variety of inflammatory mediators before the development of extensive tau-related neuropathology and brain atrophy (Leng and Edison, 2021). Accumulating evidence suggests that delirium and acute systemic infections may play a significant role in promoting or accelerating neurodegenerative disease although the mechanism for these associations is poorly understood (Walker, 2019).

During an acute infection the main trigger of inflammation is the recognition of highly conserved features of microbes (e.g., lipopolysaccharide, LPS) by “pattern-recognition receptors” (PRR) expressed by resident immune cells which include the cell-surface toll-like receptors (TLRs). Activation of TLRs initiates a conserved signaling cascade that culminates in the activation of immune cells with the release of proinflammatory cytokines (particularly TNF-α and IL-1) in the infected organ. A large amount of evidence derived from animal models has established that resident microglia in the Central Nervous System (CNS), which in healthy conditions remain in a quiescent state, express a multitude of surface receptors able to detect immune signals initiated at the periphery. Following activation, these cells initiate a neuroinflammatory reaction with the production of proinflammatory cytokines including IL-1, IL-6, and TNF-α and chemokines such as monocyte chemoattractant protein-1 able to attract monocytes from the circulation into the brain (Cerejeira et al., 2014). Animals with advanced age and/or with neurodegenerative disorders show an amplified neuroinflammatory response to acute systemic inflammation (Norden and Godbout, 2013). In animal models of chronic neurodegeneration a peripheral immune challenge has been shown to induce irreversible cell loss and progression of neurodegenerative disease (Cunningham et al., 2009). Evidence exploring the contribution of acute systemic inflammation (e.g., infection) in the pathogenesis and natural course of neurodegenerative disorders remains very limited in humans. Acute systemic inflammatory events have been associated with a 2-fold increase in the rate of cognitive decline over a 6-month period (Norden and Godbout, 2013) and patients with AD who experienced an infection and/or delirium have accelerated cognitive decline (Fong et al., 2009; Holmes et al., 2009).

Therefore, acute infection at the periphery, even without identifiable involvement of the CNS, can have a major impact in the brain and can mediate the relation between increasing age, neurodegeneration and delirium as well as the relation between delirium and cognitive deterioration at long term. Until now, the evidence relating systemic inflammation to delirium has been difficult to translate into clinical practice as the relationship between the absolute levels of inflammatory mediators at the periphery [i.e., blood or cerebrospinal fluid (CSF)] and clinical symptoms is inconsistent. While growing evidence supports the role of chronic neuroinflammatory events in neurodegenerative conditions, no studies have directly examined the neuroinflammatory response to acute systemic infection in older people with dementia. This study aims to determine the magnitude of the neuroinflammatory response triggered by an acute systemic infection at the periphery in older subjects with dementia and/or delirium.

## Methods

### Recruitment of participants

Older patients with unplanned acute admission to Internal Medicine wards between January 2018 and January 2020 with acute bacterial infection not directly involving the CNS (C-reactive protein plasma levels superior to 1 mcg/mL and requiring treatment with antibiotics) were eligible to enter the study. Participants were excluded when admitted for less than 48 h and those not able to undergo neuropsychological assessment. Other exclusion criteria were: current episode of trauma, a history of chronic inflammatory diseases, chronic use of anti-inflammatory drugs, infection of the CNS, having suffered a head trauma that resulted in a loss of consciousness or other neurological diseases, drug or alcohol addiction as well as prior chronic exposure to neurotoxic substances. A written informed consent was obtained for each participant. This study was submitted and approved by the Ethical Committee of Centro Hospitalar Universitário de Coimbra (Ethics approval Ref. CHUC-065-18).

### Clinical assessment

#### Assessment of cognitive function

All participants were assessed within 72 h of admission by a psychiatrist with the Richmond Agitation and Sedation Scale (RASS). Participants with RASS > −3 were assessed with the Confusion Assessment Method (CAM) during a formal cognitive test with the validated Portuguese version of Montreal Cognitive Assessment (MoCA) and the Mini Mental State Examination (MMSE) for illiterate participants or those with no formal education.

A diagnosis of prior dementia was obtained following a structured clinical assessment based on DSM-IV-TR criteria. Information on premorbid function was gathered from relatives (if available) or formal/informal caregivers based on the Informant Questionnaire on Cognitive Decline in the Elderly (IQCODE-SF), the Global Deterioration Scale (for examining dementia severity; GDS) and review of clinical records. Participants were assessed daily for the development of new episodes of delirium, based on all sources of information available, using RASS and CAM. The classification of patients with delirium was made if they developed at least one episode of delirium (irrespective of its severity) during hospitalization.

Participants were classified in four groups based on their cognitive status during hospitalization: (a) with delirium (b) with dementia; and (c) delirium superimposed on dementia; (d) with normal cognition (control group).

#### Demographic and medical data

We gathered information about: demographic data (age, gender, education, place of residence); current medication list, smoking habits, alcohol consumption, and previous psychiatric or neurologic diseases; severity of chronic comorbidities (Charlson Co-Morbidity scale), type of acute medical illness classified according to ICD-10; Barthel Index before hospitalization (BI); length of hospital stay and mortality at 12 months; number and severity of neuropsychiatric symptoms prior to hospital admission, using the Neuropsychiatric Inventory (NPI).

### Positron emission tomography acquisition

The [^11^C]-PK11195 PET acquisitions were performed at the Institute of Nuclear Sciences Applied to Health, University of Coimbra using a Philips Gemini GXL PET/CT scanner (Philips Medical Systems, Best, Netherlands). The participants underwent a dynamic 3-dimensional PET scan of the entire brain (90 slices, 2-mm slice sampling), preceded by a low-dose brain CT scan for attenuation correction. The dynamic [^11^C]-PK11195 image consists of 22 frames (total duration of 60 min: 4 × 30 s + 4 × 60 s + 4 × 120 s + 4 × 240 s + 6 × 300 s). The PET image acquisition sessions started immediately after the intravenous bolus injection of approximately 370MBq of [^11^C]-PK11195. To minimize movements, the head of the participants was restrained with a soft elastic tape. The PET images were reconstructed to a 128 × 128 × 90 matrix, with 2-mm isotropic voxel dimension, using the LOR RAMLA algorithm (Philips PET/CT Gemini GXL) with attenuation and scatter correction.

### Positron emission tomography image pre-processing and quantitative analysis

The pre-processing and voxelwise quantitative analysis of the [^11^C]-PK11195 PET images were implemented employing an in-house made software (Jorge et al., 2020, 2021). For each participant, a sum image in the PET native space was obtained from all the frames of the dynamic PET. The geometric transformation between the [^11^C]-PK11195 PET native space and the Montreal Neurological Institute (MNI) standard space was estimated using a template-based approach, considering an affine and a deformable model (coarse grid) based on cubic B-splines, the sum image in the PET native space as the moving image, and a sum image template in MNI space (template space MNI ICBM152) as the target reference image. The estimation of the transformations and the resampling of the images was implemented by the in-house made software, which integrates the Insight Toolkit (ITK) image analysis library.^1^

Next, all frames of the dynamic image were also registered to the MNI space using the same geometric transformation. The individual [^11^C]-PK11195 distribution volume ratio (DVR) map was estimated by applying the Logan plot method (Logan, 2000), defining the cerebellar gray matter (essentially the cerebellum without the cerebellar peduncles) as the reference region (Conen et al., 2021). For each participant, the mean DVR value of the regions-of-interest (ROI) was extracted from the individual voxelwise DVR map in the MNI space, using the Hammers’ brain atlas (Hammers et al., 2003), employing 3D Slicer 4.8.1 software (Kikinis et al., 2014).

### Statistical analyses

The analyses performed in this study followed EQUATOR reporting guidelines and were consistent with the STROBE outline for cross sectional studies. IBM SPSS Statistics (IBM, version 23) was used for demographic and clinical data analyses. Data distribution was checked by the Shapiro–Wilk test and visually. Normally distributed data were presented as mean and SD. Categorical data were presented as numbers (percentage). Test results were statistically significant for alpha < 0.05. Specifically for neuroimaging data extracted, using the aseg atlas from the FreeSurfer 6.0.0 package (Fischl et al., 2002), we extracted three regions, gray matter, white matter, and subcortical areas (ventricles included). For each of these ROIs, a voxel-wise approach was used to compare the control DVR intensities with the other groups (delirium, dementia, and delirium + dementia) in both directions. Using the MATLAB software (2021b), the function randomize two-sample unpaired *t*-test from the FSL package was used with 5,000 permutations corrected by threshold-free cluster enhancement (Smith and Nichols, 2009) and multiple comparisons. The resulting statistical maps were thresholded (corrected *p* < 0.05) and the significant voxels were located in the aparc + aseg atlas (FreeSurfer 6.0.0) (Fischl et al., 2004). We then averaged the DVR intensities into the region with the highest overlap for each participant and performed a linear regression against their assessment of cognitive function scores (CAM, MoCA-execution, MoCA-attention, MoCA-memory, MoCA-language, MOCA-orientation, IQCODE-SF, GDS, NPI). Before the regression we searched for outliers (elements higher than 3 standard deviations), and the *p*-values were corrected by multiple comparisons (Bonferroni–9 assessments).

## Results

### Sample characteristics

We originally recruited 28 participants for the study. Three subjects were excluded from PET analyses due to extreme movement artifacts and other six due to technical issues (3 from the cognitively healthy group, 3 from the Dementia group, 2 from the Delirium superimposed on dementia group, and 1 from the Delirium group). A final sample of 19 participants was obtained (Table 1).

**TABLE 1:** Socio-demographic and clinical information.

	Delirium (*n* = 4)	Dementia (*n* = 4)	Delirium superimposed on dementia (*n* = 4)	Cognitively healthy (*n* = 7)	*P-value*
Age	83.7 (2.9)	83.5 (8.7)	83.3 (6.7)	80.0 (11.7)	0.853
Gender (female)	3 (75.0)	3 (75.0)	2 (50.0)	4 (57.1)	0.736
**Education level (%)**					
No formal education	2 (50.0)	2 (50.0)	3 (75.0)	2 (28.6)	0.954
<5 years	2 (50.0)	2 (50.0)	1 (25.0)	3 (42.8)	
=5 years	0	0	0	2 (28.6)	
Nursing home care before hospitalization (n, %)	2 (50.0)	2 (50.0)	1 (25.0)	0	0.26
Barthel Index before hospitalization	70.0 (19.7)	58.33 (28.8)	50.1 (19.49)	91.0 (8.2)	0.321
MMSE at discharge	6.00 (*N* = 1)	12.00 (*N* = 1)	10.00 (*N* = 1)	NA	NA
MoCA at discharge	6.5 (4.95)	6.00 (1.73)	5.3 (2.01)	19.9 (4.62)	**0.021**
**Type of infection**					
Respiratory	4 (100.0)	2 (50.0)	2 (50.0)	3 (42.9)	0.158
Urinary	0	0	2 (50.0)	3 (42.9)	
Other	0	2 (50.0)	0	1 (14.2)	
Pain score	4.0 (2.8)	2.8 (2.01)	4.7 (2.1)	2.5 (2.6)	0.570
Length of stay	14.0 (3.6)	8.0 (3.6)	13.0 (9.3)	6.3 (1.5)	**0.07**
Mortality at 18 months	1 (25)	1 (25)	1 (25)	0	0.141
Leucocytes	12.83 (7.8)	7.7 (1.0)	8.75 (4.55)	9.06 (4.24)	0.535
Neutrophiles	9.51 (8.6)	3.57 (2.45)	2.77 (1.98)	4.93 (4.19)	0.275
Monocytes	0.69 (0.22)	0.31 (0.24)	0.42 (0.37)	0.58 (0.45)	0.521
C-reactive protein	3.54 (3.12)	4.11 (3.02)	5.32 (0.32)	13.9 (11.4)	0.145

Bold numbers represent *p* values lower than 0.05.

### Relation between cognitive status and distribution volume ratio in positron emission tomography

**FIGURE 1:**
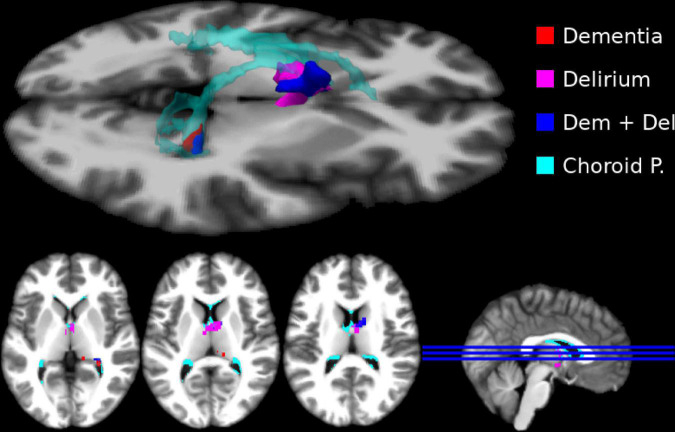
Subcortical regions where the cognitively healthy (CH) sample presented higher distribution volume ratio (DVR). Here we show significant subcortical differences (thresholded at *p* < 0.05 FEW corrected) in DVR intensity per group (CH > Delirium–magenta; CH > Dementia–red; CH > Delirium + Dementia–blue). We show an overlap between the regions that presented significant DVR differences and choroid plexus (cyan). No other effects for experimental groups were significant.

We found no significant group differences for gray-matter and white-matter analysis (both directions) (Supplementary material). In the subcortical analysis, we found that the cognitively healthy group presented regions with significantly higher DVR intensity (0.856) than the other groups in the choroid plexus (Delirium = 0.601; Dementia = 0.614; Delirium Superimposed on Dementia = 0.559; between groups *p* = 0.012) (Figure 1).

#### Positron emission tomography choroid plexus microglia activation and clinical and cognitive status

**FIGURE 2:**
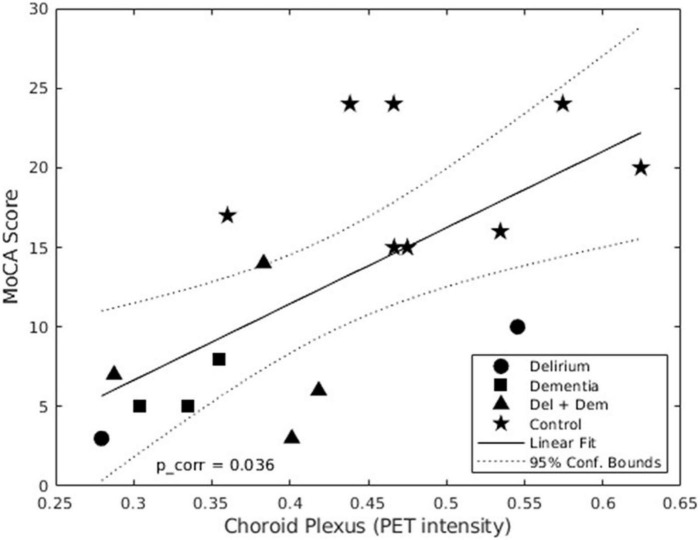
Linear regression of mean distribution volume ratio (DVR) in choroid plexus and MoCA scores. In this graph we show that there is a positive linear relation between the MoCA score and the mean choroid plexus DVR. This means that the highest DVR intensity in the choroid plexus corresponds to a better cognitive performance by the patients enrolled in the study.

No outliers were found in the nine linear regression analyzes performed between choroid plexus DVR and clinical measures. The association between mean choroid plexus DVR and MoCA score was the only one that showed a significant linear correlation (*p* < 0.036). Its Pearson’s correlation coefficient was 0.66 and the adjusted *R*^2^ value was 0.4, so 40% of the variation in MoCA attention scores can be explained by the model containing only choroid plexus DVR levels. The scatter plot of standardized predicted values vs. standardized residuals, showed that the data met the assumptions of homogeneity of variance and linearity and the residuals were approximately normally distributed (Figure 2).

## Discussion

Immune dysregulation is being increasingly recognized as a hallmark of neurodegenerative disorders and has been also implicated in delirium pathophysiology. Yet, it remains unclear to what extent the neuroinflammatory response to an acute systemic infection is altered in patients with cognitive dysfunction (dementia and/or delirium). In this study we determined the level of neuroinflammation using PET with a TSPO ligand in older participants hospitalized with an acute systemic infection. We found that cognitively healthy participants had higher DVR intensity than participants with cognitive impairment (dementia and/or delirium) in subcortical regions (choroid plexus).

In AD patients, chronic neuroinflammation is higher in cortical regions associated with early amyloid deposition and during AD progression the intensity of neuroinflammation negatively correlates with MMSE scores in the parietal region (Bradburn et al., 2019). According to preclinical data, higher baseline levels of neuroinflammation in participants with prior dementia are expected to promote a more intense acute neuroinflammatory reaction to acute systemic infection eventually triggering an episode of delirium (Cerejeira et al., 2010). However, we found that participants with dementia and/or delirium had a similar DVR intensity in the cerebral cortex than cognitively healthy participants. Moreover, we observed a higher DVR intensity in cognitively healthy participants in choroid plexus suggesting that participants with dementia and/or delirium have a weaker neuroinflammatory response to acute systemic infection. In line with our results, systemic infection has been associated, in AD patients, with downregulation of a range of pro-inflammatory markers, upregulated expression of the anti-inflammatory genes IL4R and CHI3L1, reduced microglial phagocytic activity as well as reduced T cell recruitment in the brain (Rakic et al., 2018). Importantly, two types of microglial response to immune challenges have been described, training and tolerance, which enhance or suppress subsequent inflammation, respectively. These different microglial responses are epigenetically determined and it is possible that the neuropathology underlying dementia induces an immunosuppressive environment that incapacitates the immune system to respond appropriately to systemic infection. This would also explain our finding of a higher DVR intensity in the choroid plexus of cognitively healthy participants following a growing body of evidence showing that the blood-cerebrospinal fluid barrier plays a crucial role in the spread of inflammatory reactions from the periphery to the CNS (Solár et al., 2020). Thus, in animal models, acute LPS injection triggers a rapid and transient alteration in the choroid plexus gene expression profile encoding for proteins belonging to the NF-kB, MAPK, STAT-JAK, and IRFs signaling pathways returning to basal levels after 3 days (Marques et al., 2007). The DVR intensity positively correlated with global cognitive function suggesting that cognitive status, significantly impaired in the groups with dementia and/or delirium, are associated with a less robust neuroinflammatory response in the choroid plexus.

A weakness of our study is that the ligand used in PET can only indicate the activation level of the regional analysis, rather, they are unable to differentiate between pro and anti-inflammatory states of microglia and astrocytes. Moreover, although we included participants within the first 72 h of hospital admission, the acute and transient nature of the neuroinflammatory response may have not been adequately captured during the PET scan acquisition. Also, the restricted recruitment of older participants hospitalized with an acute infection and able to be cognitively assessed has resulted in a small sample and precluded the detailed characterization of the dementia subtype which is a major limitation of the current study. This affected the robustness of comparison across the different groups (i.e., delirium, dementia and delirium superimposed on dementia) and restricted the power of the regional analysis. Thus, future investigations should clarify if different disorders underlying dementia can have distinct neuroinflammatory profiles in response to acute systemic infection.

In summary, our study suggests that dementia and/or delirium is associated with a reduced neuroinflammatory response to acute systemic infection which can be the result of an immunosuppressive brain environment.

## Data availability statement

The raw data supporting the conclusions of this article will be made available by the authors, without undue reservation.

## Ethics statement

The studies involving human participants were reviewed and approved by Ethical Committee of Centro Hospitalar e Universitário de Coimbra (Ethics approval Ref. CHUC-065-18). The patients/participants provided their written informed consent to participate in this study.

## Author contributions

ARS, PR, and JC formulated the research question, designed the study, and drafted the work. ARS, PR, and ALC contributed to the acquisition, analysis, or interpretation of data collected. AP contributed to the analysis and interpretation of neuroimaging data and together with ARS, performed the statistical analysis. IS, IB, ALC, and JC revised the work critically and together with ARS, PR, ALC and AP, contributed to the final approval of the version to be published. All authors contributed with different roles in the present work, agreed with the presented findings, and approved the submitted version.

## Footnotes

1
https://itk.org/
