# Supplementary material for: In vivo molecular imaging of the neuroinflammatory response to peripheral acute bacterial infection in older patients with cognitive dysfunction: A cross-sectional controlled study
Source: Front Aging Neurosci. 2022 Sep 7;14:984178. doi: 10.3389/fnagi.2022.984178 (PMC9491091; doi:10.3389/fnagi.2022.984178)
Supplement: Supplementary file 1 [file Data_Sheet_1.DOCX]

**Supplementary data**

| **Supplementary Table 1.** Mean value of the distribution volume ratio (DVR) in the regions-of-interest (ROI) extracted from the individual voxelwise map using the Hammers' brain atlas**.** Significance values correspond to multiple Kruskal-Wallis Tests.  Group 1: delirium; Group 2: dementia; Group 3: delirium superimposed on dementia; Group 4: cognitively healthy controls | | | | | |
| --- | --- | --- | --- | --- | --- |
|  | **Mean DVR by group** | | | | **p value** |
| **Region of interest (ROI)** | 1 | 2 | 3 | 4 |  |
| Left Hippocampus | 0.752 | 0.767 | 0.741 | 0.830 | 0.242 |
| Right Hippocampus | 0.740 | 0.783 | 0.739 | 0.823 | 0.200 |
| Left Amygdala | 0.746 | 0.797 | 0.799 | 0.797 | 0.718 |
| Right Amygdala | 0.766 | 0.834 | 0.819 | 0.789 | 0.652 |
| Left anterior medial temporal lobe | 0.889 | 0.868 | 0.836 | 0.863 | 0.911 |
| Right anterior medial temporal lobe | 0.825 | 0.851 | 0.804 | 0.893 | 0.305 |
| Left anterior lateral temporal lobe | 0.710 | 0.797 | 0.684 | 0.751 | 0.234 |
| Right anterior lateral temporal lobe | 0.725 | 0.789 | 0.757 | 0.771 | 0.352 |
| Left parahippocampal gyrus | 0.845 | 0.857 | 0.868 | 0.842 | 0.871 |
| Right parahippocampal gyrus | 0.808 | 0.830 | 0.834 | 0.880 | 0.308 |
| Left superior temporal gyrus | 0.724 | 0.751 | 0.724 | 0.753 | 0.810 |
| Right superior temporal gyrus | 0.725 | 0.760 | 0.733 | 0.768 | 0.663 |
| Left inferior middle temporal gyrus | 0.834 | 0.834 | 0.806 | 0.835 | 0.904 |
| Right inferior middle temporal gyrus | 0.809 | 0.863 | 0.802 | 0.864 | 0.223 |
| Left fusiform gyrus | 0.896 | 0.837 | 0.855 | 0.862 | 0.647 |
| Right fusiform gyrus | 0.829 | 0.885 | 0.843 | 0.919 | 0.120 |
| Left cerebellum | 0.862 | 0.859 | 0.876 | 0.874 | 0.922 |
| Right cerebellum | 0.864 | 0.890 | 0.897 | 0.891 | 0.774 |
| Left brainstem | 0.808 | 0.849 | 0.832 | 0.849 | 0.639 |
| Right brainstem | 0.791 | 0.855 | 0.879 | 0.842 | 0.151 |
| Left insula | 0.704 | 0.722 | 0.669 | 0.713 | 0.609 |
| Right insula | 0.722 | 0.723 | 0.702 | 0.728 | 0.933 |
| Left lateral occipital lobe | 0.812 | 0.821 | 0.780 | 0.873 | 0.243 |
| Right lateral occipital lobe | 0.823 | 0.871 | 0.806 | 0.867 | 0.473 |
| Left anterior cingulate gyrus | 0.616 | 0.714 | 0.608 | 0.690 | 0.168 |
| Right anterior cingulate gyrus | 0.666 | 0.713 | 0.607 | 0.682 | 0.158 |
| Left posterior cingulate gyrus | 0.744 | 0.779 | 0.728 | 0.766 | 0.808 |
| Right posterior cingulate gyrus | 0.764 | 0.788 | 0.683 | 0.762 | 0.252 |
| Left middle frontal gyrus | 0.673 | 0.695 | 0.653 | 0.749 | 0.447 |
| Right middle frontal gyrus | 0.663 | 0.708 | 0.664 | 0.750 | 0.288 |
| Left posterior temporal lobe | 0.771 | 0.770 | 0.749 | 0.799 | 0.715 |
| Right posterior temporal lobe | 0.773 | 0.799 | 0.741 | 0.814 | 0.158 |
| Left inferior lateral parietal lobe | 0.730 | 0.706 | 0.690 | 0.772 | 0.258 |
| Right inferior lateral parietal lobe | 0.740 | 0.755 | 0.705 | 0.763 | 0.576 |
| Left caudate | 0.400 | 0.408 | 0.394 | 0.476 | 0.241 |
| Right caudate | 0.461 | 0.512 | 0.465 | 0.545 | 0.067 |
| Left accumbens | 0.657 | 0.676 | 0.595 | 0.733 | 0.100 |
| Right accumbens | 0.773 | 0.785 | 0.698 | 0.800 | 0.433 |
| Left putamen | 0.846 | 0.772 | 0.747 | 0.786 | 0.454 |
| Right putamen | 0.836 | 0.794 | 0.767 | 0.815 | 0.498 |
| Left thalamus | 0.682 | 0.646 | 0.658 | 0.763 | 0.060 |
| Right thalamus | 0.709 | 0.669 | 0.693 | 0.799 | 0.090 |
| Left pallidum | 0.812 | 0.800 | 0.745 | 0.810 | 0.597 |
| Right pallidum | 0.862 | 0.822 | 0.784 | 0.847 | 0.536 |
| Left corpus callosum | 0.509 | 0.518 | 0.484 | 0.538 | 0.750 |
| Right corpus callosum | 0.470 | 0.479 | 0.428 | 0.509 | 0.347 |
| Left lateral temporal ventricle | 0.320 | 0.312 | 0.337 | 0.396 | 0.397 |
| Right lateral temporal ventricle | 0.330 | 0.339 | 0.315 | 0.427 | 0.089 |
| Left 3rd ventricle | 0.456 | 0.520 | 0.540 | 0.638 | 0.050 |
| Right 3rd ventricle | 0.445 | 0.534 | 0.546 | 0.637 | 0.080 |
| Left precentral gyrus | 0.734 | 0.690 | 0.682 | 0.729 | 0.656 |
| Right precentral gyrus | 0.716 | 0.725 | 0.669 | 0.728 | 0.558 |
| Left gyrus rectus | 0.764 | 0.744 | 0.770 | 0.759 | 0.578 |
| Right gyrus rectus | 0.799 | 0.779 | 0.759 | 0.798 | 0.798 |
| Left orbitofrontal gyrus | 0.807 | 0.851 | 0.772 | 0.829 | 0.531 |
| Left orbitofrontal gyrus | 0.777 | 0.831 | 0.804 | 0.868 | 0.334 |
| Left inferior frontal gyrus | 0.732 | 0.730 | 0.691 | 0.774 | 0.507 |
| Right inferior frontal gyrus | 0.709 | 0.766 | 0.732 | 0.776 | 0.661 |
| Left superior frontal gyrus | 0.682 | 0.685 | 0.632 | 0.723 | 0.330 |
| Right superior frontal gyrus | 0.695 | 0.691 | 0.635 | 0.711 | 0.283 |
| Left postcentral gyrus | 0.721 | 0.687 | 0.674 | 0.735 | 0.589 |
| Right postcentral gyrus | 0.711 | 0.703 | 0.659 | 0.729 | 0.532 |
| Left superior parietal gyrus | 0.748 | 0.743 | 0.719 | 0.777 | 0.550 |
| Right superior parietal gyrus | 0.737 | 0.733 | 0.692 | 0.749 | 0.496 |
| Left lingual gyrus | 0.852 | 0.840 | 0.852 | 0.902 | 0.340 |
| Right lingual gyrus | 0.872 | 0.874 | 0.839 | 0.882 | 0.665 |
| Left cuneus | 0.854 | 0.881 | 0.859 | 0.909 | 0.608 |
| Right cuneus | 0.878 | 0.897 | 0.870 | 0.889 | 0.934 |
